# *CDK4/6* Inhibitors in Breast Cancer: Clinical Applications, Translational Insights, and Future Directions

**DOI:** 10.3390/cancers18152376

**Published:** 2026-07-23

**Authors:** Mengying Guan, Hua Hao

**Affiliations:** 1 Department of Breast Surgery, Yangpu Hospital, School of Medicine, Tongji University, Shanghai 200090, China; 2480166@tongji.edu.cn; 2 Department of Pathology, Yangpu Hospital, School of Medicine, Tongji University, Shanghai 200090, China

**Keywords:** *CDK4/6* inhibitors, breast cancer, HR+/HER2−, resistance mechanisms, precision medicine

## Abstract

Cyclin-dependent kinase 4/6 inhibitors have fundamentally changed the management of hormone receptor-positive, human epidermal growth factor receptor 2-negative breast cancer. However, these drugs are not interchangeable and the field is moving away from the notion of a uniform “class effect.” In early breast cancer, adjuvant abemaciclib and ribociclib improve invasive disease-free survival in patients at a high risk of recurrence, whereas palbociclib does not. This difference likely stems from agent-specific pharmacological profiles, differences in trial design, and patient selection, rather than simply dosing nuances. In metastatic breast cancer, all three agents prolong progression-free survival when combined with endocrine therapy, but only ribociclib and potentially abemaciclib have shown an overall survival advantage. In addition, resistance remains a major obstacle in clinical practice. We propose that resistance mechanisms can be meaningfully grouped into two categories: target-driven (e.g., *RB1* loss, *CDK6* amplification) and bypass-driven (e.g., *ESR1* mutations, PI3K/AKT pathway activation, *APOBEC3*-mediated mutagenesis). Distinguishing between these classes helps in the design of rational sequencing algorithms and combinatorial regimens. Emerging strategies, such as next-generation protein degraders, oral selective estrogen receptor degraders, antibody–drug conjugates, and inhibition of autophagy, are promising methods for overcoming resistance. Moving forward, the greatest need in breast cancer treatment will be not simply developing additional agents but using current therapies more intelligently by refining biomarker-guided patient selection, tailoring treatment duration, and ensuring broad global access.

## Simple Summary

Cyclin-dependent kinase 4/6 (*CDK4/6*) inhibitors have transformed the treatment landscape for hormone receptor-positive, human epidermal growth factor receptor 2-negative (HR+/HER2−) breast cancer. This review examines the clinical development of palbociclib, ribociclib, and abemaciclib across both early-stage and metastatic settings, revealing clinically meaningful differences in efficacy that challenge the notion of a uniform class effect. We discuss established and emerging resistance mechanisms, including *RB1* loss, *ESR1* mutations, and *APOBEC3*-mediated mutagenesis, and propose a conceptual framework that classifies resistance as either target-driven or bypass-driven to inform rational treatment sequencing. Emerging therapeutic strategies—including next-generation protein degraders (PROTACs), oral selective estrogen receptor degraders, antibody–drug conjugates, and autophagy inhibitors—are highlighted as promising approaches to overcome or circumvent resistance. Looking forward, the greatest advances will likely come not from developing additional agents alone, but from more intelligent use of existing therapies through refined biomarker-guided patient selection, optimized treatment sequencing, and equitable global access.

## 1. Introduction

This narrative review was conducted through systematic searches of PubMed/MEDLINE, Embase, Cochrane Library, and ClinicalTrials.gov, covering publications from January 2000 to June 2026. Search terms were combined using Boolean operators. The core search strategy was defined as: (“CDK4/6 inhibitors” OR palbociclib OR ribociclib OR abemaciclib) AND (“breast cancer” OR “breast carcinoma”) AND (“hormone receptor-positive” OR “HR+” OR “HR-positive”) AND (“HER2-negative” OR “HER2−”). Additional targeted sub-searches, all restricted to the above core framework to exclude irrelevant studies, were performed focusing on drug resistance mechanisms, biomarker detection, novel therapeutic strategies, and clinical trial evidence: (“resistance mechanism” OR “drug resistance”) AND (“RB1” OR “ESR1” OR “PIK3CA” OR “PTEN” OR “*CDK6* amplification” OR “APOBEC3”); (“liquid biopsy” OR “circulating tumor DNA” OR “ctDNA”); (“PROTAC” OR “oral SERD” OR “antibody-drug conjugate” OR “autophagy inhibitor”); and (“clinical trial” OR “randomized controlled trial” OR “phase III”). Literature screening priority was given to phase III randomized controlled trials, followed by phase II trials, real-world evidence studies, and preclinical investigations. Only English-language publications were included. Guidelines from the National Comprehensive Cancer Network (NCCN) and the European Society for Medical Oncology (ESMO) were consulted to ensure alignment with current standard-of-care recommendations.

Hormone receptor-positive, human epidermal growth factor receptor 2-negative (HR+/HER2−) breast cancer accounts for approximately 70% of all breast cancer cases [1]. Its biology is predominantly governed by the cyclin D–cyclin-dependent kinase 4/6 (*CDK4/6*)–retinoblastoma (RB) axis, which regulates the progression from the G1 to the S phase of the cell cycle. Estrogen receptor (ER) signaling promotes cyclin D1 expression, which in turn activates CDK4/6, resulting in RB phosphorylation, E2F release, and subsequent cell proliferation [2].

**Figure 1:**
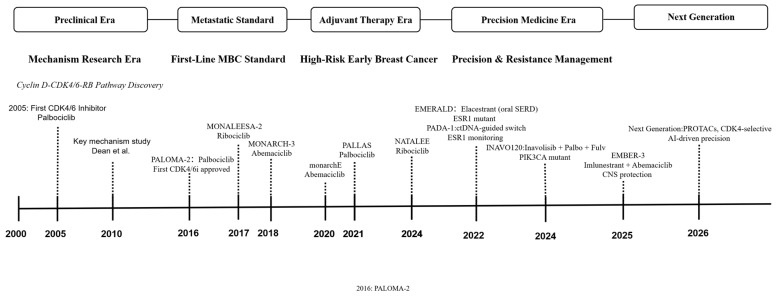
Evolution of *CDK4/6* inhibitors in breast cancer (2000–2026). The timeline illustrates the progression from preclinical mechanism research to current precision-medicine and next-generation strategies. Key milestones include: the discovery of the cyclin D–*CDK4/6*–retinoblastoma axis and early preclinical development of the first *CDK4/6* inhibitor palbociclib (PD-0332991) [2,9,10]; the establishment of first-line metastatic standard-of-care through pivotal phase III trials—PALOMA-2 (palbociclib) [3], MONALEESA-2 (ribociclib) [4], and MONARCH-3 (abemaciclib) [5]; the evaluation of adjuvant therapy in high-risk early breast cancer via monarchE (abemaciclib) [6], PALLAS (palbociclib) [8], and NATALEE (ribociclib) [7]; the emergence of precision-medicine strategies including the oral selective estrogen receptor degrader (SERD) elacestrant in EMERALD for *ESR1*-mutant disease [11], the ctDNA-guided treatment-switch strategy in PADA-1 [12], and the brain-penetrant oral SERD imlunestrant plus abemaciclib in EMBER-3 [13]; and next-generation investigational approaches such as proteolysis-targeting chimeras (PROTACs) [14,15], *CDK4*-selective inhibitors [16], and artificial intelligence–driven predictive models [17,18]. Abbreviations: *CDK4/6*, cyclin-dependent kinase 4/6; SERD, selective estrogen receptor degrader; ctDNA, circulating tumor DNA; CNS, central nervous system; PROTAC, proteolysis-targeting chimera.

*CDK4/6* inhibitors were initially established as effective in metastatic settings. In the PALOMA-2 trial, the addition of palbociclib to letrozole prolonged the median progression-free survival (PFS) by 10 months compared with letrozole alone (hazard ratio, 0.58; *p* < 0.001) [3]. Comparable benefits were observed for ribociclib in the MONALEESA-2 [4] trial and for abemaciclib in the MONARCH-3 [5] trial. These results led to the evaluation of CDK4/6 inhibitors as adjuvant therapies for early-stage breast cancer. However, a key divergence soon became apparent. The monarchE (abemaciclib) [6] and NATALEE (ribociclib) [7] trials demonstrated significantly improved invasive disease-free survival (iDFS), whereas the PALLAS trial (palbociclib) did not [8]. Real-world evidence from the SEER database further corroborates the survival advantage associated with *CDK4/6* inhibitor therapy [1]. This discrepancy does not invalidate the *CDK4/6* inhibitor class as a whole, but rather it underscores the clinical and biological importance of agent-specific differences (Figure 1).

These discordant outcomes across different agents and disease stages raise three fundamental questions that frame the present review: (i) why adjuvant efficacy cannot be reliably extrapolated from metastatic therapeutic responses; (ii) how the SONIA trial redefines first- and second-line endocrine/CDK4/6 sequencing strategies; and (iii) which resistance signatures are sufficiently validated to support routine biomarker-guided treatment, as opposed to preclinical exploratory mechanisms. We advance three core clinical propositions that directly address these unmet clinical needs: First, metastatic anti-tumor activity fails to predict adjuvant recurrence reduction, as demonstrated by palbociclib’s contradictory trial performance. Second, resistance phenotypes can be stratified into target-driven and bypass-driven subtypes to inform rational combination regimens. Third, substantial clinical gains will derive from optimized treatment sequences and ctDNA-based adaptive therapy, instead of new single-agent inhibitors. This review provides actionable clinical insights by differentiating guideline-applicable evidence from unconfirmed preclinical observations.

## 2. Early Breast Cancer

This chapter analyzes neoadjuvant and adjuvant clinical data, focusing on the heterogeneous efficacy of distinct *CDK4/6* inhibitors to unpack why metastatic activity cannot translate to early-stage outcomes.

### 2.1. Neoadjuvant Therapy

Neoadjuvant trials offer a direct window into the translational gap between short-term pharmacodynamic suppression and long-term recurrence reduction, clarifying whether rapid on-treatment biomarker changes can predict durable adjuvant benefit. In the PALLET trial, 307 postmenopausal women with ER-positive primary breast cancer were randomly assigned to receive letrozole alone or in combination with palbociclib. The proportion of patients achieving complete cell cycle arrest (CCCA; Ki-67 ≤ 2.7%) was markedly greater with the combination therapy than with the single agent (90% vs. 59%; odds ratio [OR], 6.83; 95% confidence interval [CI], 3.12–14.98; *p* < 0.001), while pathological complete response (pCR) rates remained low in both groups (3.3% vs. 1.1%, *p* = 0.43) [19].

The NeoPalAna study also found that adding palbociclib to anastrozole increased the CCCA from 26% to 87% (*p* < 0.001) without any pCRs [20]. In the CARABELA trial, 12 months of letrozole plus abemaciclib was compared to chemotherapy in patients with highly proliferative (Ki-67 ≥ 20%) HR+/HER2− EBC; residual cancer burden 0–I rates were 13% vs. 18%, failing to show equivalence [21]. A meta-analysis of eight randomized controlled trials (RCTs) corroborated the improvement in CCCA, but not in pCR, with neoadjuvant *CDK4/6* inhibitors [22].

It is important to distinguish between pharmacodynamic biomarker suppression (such as Ki-67 reduction and complete cell cycle arrest) and demonstrated long-term clinical benefit. While these biomarker changes indicate biological activity of *CDK4/6* inhibitors, they have not been validated as surrogates for event-free or overall survival endpoints. The absence of pCR with *CDK4/6* inhibitor-based neoadjuvant regimens reflects their primarily cytostatic (rather than cytotoxic) mechanism of action, which suppresses cell proliferation without inducing apoptosis. In the PALLET trial, the addition of palbociclib decreased the levels of cleaved poly (ADP-ribose) polymerase (c-PARP), reflecting reduced cell death [19]. Therefore, in patients with lower proliferative tumors (Ki-67 < 30% or recurrence score < 26)—where chemotherapy benefit is limited and toxicity is substantial—neoadjuvant *CDK4/6* inhibition may offer adequate disease control with a more favorable safety profile as a de-escalation strategy, particularly when the goal is surgical resectability rather than pathological complete response [21]. A SEER analysis of 21,299 patients with stage II–III HR+/HER2− breast cancer associated NACT with inferior survival compared with adjuvant chemotherapy [23], though this is confounded by selection bias and does not prove chemotherapy inferiority. These data nonetheless underscore the need for better-tolerated neoadjuvant strategies, indirectly supporting the exploration of *CDK4/6* inhibitor-based targeted approaches in patients with limited expected chemotherapy benefit.

### 2.2. Adjuvant Therapy

While neoadjuvant analyses illustrate the gap between short-term pharmacodynamic changes and long-term recurrence control, adjuvant trials add another critical dimension: efficacy varies drastically among individual agents due to drug characteristics, administration schedules and patient risk stratification.

Adjuvant benefits differ across agents. The monarchE trial randomized 5637 high-risk, node-positive patients to receive abemaciclib (150 mg twice daily for two years) plus ET or ET alone. At five years, iDFS rates were 83.6% vs. 76.0% (hazard ratio, 0.680; 95% CI, 0.599–0.772). Distant relapse-free survival was also high in patients who received abemaciclib with ET (hazard ratio, 0.675; 95% CI, 0.588–0.774) [6]. The NATALEE trial enrolled 5101 patients with stage II–III HR+/HER2− EBC, including selected node-negative cases with high-risk features, to receive ribociclib (400 mg daily, three weeks on/one week off for three years) plus a nonsteroidal aromatase inhibitor (NSAI). At four years, iDFS was 88.5% vs. 83.6% (hazard ratio, 0.72; 95% CI, 0.61–0.84; *p* < 0.0001) [7].

By comparison, the PALLAS trial evaluated palbociclib (125 mg/day, three weeks on/one week off for two years) in 5760 patients with stage II–III disease and demonstrated no improvement in iDFS (five-year iDFS 80.7% vs. 80.3%; hazard ratio, 0.98; 95% CI, 0.84–1.14) [8]. In addition, a prespecified subgroup analysis of patients with stage IIA disease failed to show benefit (four-year iDFS 92.9% vs. 92.1%; hazard ratio, 0.75; 95% CI, 0.48–1.19) [24]. Similarly, the PENELOPE-B trial, which administered 13 cycles of palbociclib to patients with residual disease following NACT, did not meet its primary endpoint (four-year iDFS 73.0% vs. 72.4%; hazard ratio, 0.93; 95% CI, 0.74–1.17) [25]. A subsequent meta-analysis showed that adjuvant *CDK4/6* inhibitors as a class improve iDFS (hazard ratio, 0.81; 95% CI, 0.68–0.97) but not overall survival (OS), and highlighted drug-specific differences [22].

The reasons for palbociclib’s lack of adjuvant efficacy remain unclear. An exposure-efficacy analysis linked suboptimal drug exposure and early discontinuation to poorer outcomes in PALLAS, although this explanation seems the least plausible given that dose reductions were permitted yet still showed no benefit [26]. Additional factors include: (i) dosing schedule differences (continuous abemaciclib vs. intermittent palbociclib/ribociclib); (ii) dose-modification rules and tolerability; (iii) population heterogeneity across trials; (iv) the reduced ribociclib dose in NATALEE (400 mg vs. 600 mg in metastatic disease); (v) exclusion of tamoxifen co-administration in NATALEE; and (vi) insufficient follow-up to detect differential overall survival. It is important to acknowledge that the discordance between metastatic and adjuvant outcomes represents a post hoc clinical observation rather than an established biological framework [27]. Variations in *CDK4*/*CDK6* selectivity may contribute, but no single hypothesis fully explains these negative results. Most likely, multiple factors interact; the practical message is that palbociclib should not be used as an adjuvant.

### 2.3. Clinical Considerations

#### 2.3.1. Defining High Risk

In the monarchE trial, high risk was defined as having ≥4 positive lymph nodes, or 1–3 positive nodes combined with at least one of the following: tumor size ≥ 5 cm, grade 3 histology, or Ki-67 ≥ 20% [6]. The NATALEE trial enrolled patients with stage IIB–III disease and a subset of stage IIA patients with additional risk factors [7]. Real-world analyses indicate that 11–25% of HR+/HER2− EBC cases fulfill monarchE eligibility, whereas 31–43% satisfy NATALEE criteria [28].

#### 2.3.2. Treatment Duration

A longer treatment course (two years with abemaciclib and three years with ribociclib) seems to confer a greater benefit than the two-year regimen of palbociclib [8,25]. It is still uncertain whether this advantage is primarily due to treatment duration, intrinsic pharmacological differences between agents, or variations in the enrolled patient populations.

#### 2.3.3. Toxicity Profiles

Abemaciclib toxicities included grades 3–4 neutropenia that occurred in 20% of patients, diarrhea in 8%, and venous thromboembolism in 2.5% compared with 0.6% for ET alone. Ribociclib toxicities included grades 3–4 neutropenia (44%) and elevated liver transaminase levels (9%). Palbociclib toxicities included grades 3–4 neutropenia in 61% of patients, resulting in treatment discontinuation by 27% of patients [6,7,8,28]. Therefore, careful and proactive dose adjustments are essential. A network meta-analysis demonstrated a higher risk of neutropenia with palbociclib and ribociclib than with abemaciclib [29].

#### 2.3.4. Adherence and Quality of Life

Discontinuation rates were 42% in the PALLAS, 31% in the monarchE, and 36% in the NATALEE trials [6,7,8]. Health-related quality of life results from the NATALEE trial showed stable scores across all domains of the European Organization for Research and Treatment of Cancer QLQ-C30, indicating good overall tolerability [30].

## 3. Metastatic Breast Cancer

Having examined the divergent adjuvant results and their clinical implications, we now turn to the metastatic setting where *CDK4/6* inhibitors were first established as effective.

### 3.1. First-Line Therapy

Three phase III trials established *CDK4/6* inhibitors combined with ET as the first-line standard of care for HR+/HER2− metastatic breast cancer (MBC). In the PALOMA-2 (palbociclib + letrozole) trial, median PFS increased from 14.5 to 24.8 months (hazard ratio, 0.58; 95% CI, 0.46–0.72; *p* < 0.001), but the final OS analysis did not demonstrate a significant improvement (37.5 vs. 34.5 months, *p* = 0.281) [3]. In the MONALEESA-2 (ribociclib + letrozole) trial, median PFS was 25.3 vs. 16.0 months (hazard ratio, 0.56; 95% CI, 0.43–0.72; *p* < 0.001), with a significant OS advantage (63.9 vs. 51.4 months; hazard ratio, 0.76; 95% CI, 0.63–0.93; *p* = 0.008) [4]. The MONARCH-3 (abemaciclib + NSAI) trial reported a median PFS of 28.2 vs. 14.8 months (hazard ratio, 0.54; 95% CI, 0.41–0.72; *p* < 0.001), and OS numerically favored abemaciclib (66.8 vs. 53.7 months; hazard ratio, 0.804; *p* = 0.066) [5].

A meta-analysis of 12 RCTs demonstrated improved PFS (hazard ratio, 0.57; 95% CI, 0.53–0.62) and OS (hazard ratio, 0.81; 95% CI, 0.73–0.89) with *CDK4/6* inhibitors in combination with ET [31]. It is important to acknowledge that direct cross-trial comparisons of OS across PALOMA-2, MONALEESA-2, and MONARCH-3 are methodologically limited by differences in trial design, including prior chemotherapy eligibility, proportions of de novo metastatic disease, randomization ratios, stratification factors, and rates of missing OS data. Therefore, any apparent differences in OS outcomes between agents should be interpreted with caution, and PFS remains the most robust comparable endpoint across trials. Subgroup analyses showed benefits in older patients (>70 years; pooled median PFS 18.6 vs. 10.9 months; hazard ratio, 0.56) and patients with visceral metastases, although the presence of liver metastases was associated with poorer outcomes [32,33]. A pooled analysis focusing on elderly patients further supported the feasibility of this regimen with careful monitoring [34].

Real-world evidence is consistent with clinical trial results. In Serbia, first-line ribociclib treatment achieved a median PFS of 30 months [33]. In a Korean cohort of 1115 patients, *CDK4/6* inhibitors combined with ET significantly prolonged PFS compared with ET alone (hazard ratio, 0.64; 95% CI, 0.55–0.75; *p* < 0.001) [35]. A Japanese study reported a median PFS of 19.3 months in patients who received palbociclib plus ET [36]. A Canadian real-world analysis comparing palbociclib and ribociclib showed that the two agents had similar efficacy [37]. Additionally, a propensity score-adjusted study in 312 patients confirmed the PFS advantage of combination therapy (median 18.2 vs. 11.4 months; hazard ratio, 0.61; 95% CI, 0.47–0.79) [38].

### 3.2. Second-Line and Subsequent Therapy

Having examined the consistent first-line progression-free survival benefits and their real-world validation, we now turn to therapeutic sequencing for second- and later-line disease, where acquired resistance dictates clinical decision-making.

#### 3.2.1. Second-Line Standard Regimens

The standard second-line approach following progression on a *CDK4/6* inhibitor combined with an AI is fulvestrant-based endocrine therapy, with or without continued *CDK4/6* inhibition. Three pivotal phase III trials established the efficacy of *CDK4/6* inhibitors combined with fulvestrant in the second-line setting. The MONARCH-2 trial randomized 669 patients with disease progression after prior ET to receive abemaciclib plus fulvestrant vs. placebo plus fulvestrant. The combination significantly improved median PFS (16.4 vs. 9.3 months; hazard ratio, 0.55; 95% CI, 0.45–0.68; *p* < 0.001) and median OS (46.7 vs. 37.3 months; hazard ratio, 0.76; 95% CI, 0.61–0.96; *p* = 0.014) [39]. Similarly, the PALOMA-3 trial demonstrated that palbociclib plus fulvestrant improved median PFS compared with placebo plus fulvestrant (9.2 vs. 3.8 months; hazard ratio, 0.42; 95% CI, 0.32–0.56; *p* < 0.001) in 521 patients who had relapsed or progressed after prior ET, although the OS difference did not reach statistical significance (median 34.9 vs. 28.0 months; hazard ratio, 0.81; 95% CI, 0.65–1.00; *p* = 0.022, not meeting the prespecified alpha level) [40]. The MONALEESA-3 trial evaluated ribociclib plus fulvestrant in 726 patients with no or limited prior ET, showing improved median PFS (20.5 vs. 12.8 months; hazard ratio, 0.59; 95% CI, 0.48–0.73; *p* < 0.001) and median OS (67.6 vs. 51.8 months; hazard ratio, 0.67; 95% CI, 0.50–0.90; *p* = 0.004) [41].

#### 3.2.2. The SONIA Trial and Sequencing Debate

These trials collectively established *CDK4/6* inhibitor plus fulvestrant as the standard second-line regimen. However, an important clinical question is whether *CDK4/6* inhibitors should be used in the first or second line. The phase III SONIA trial [42] addressed this question by randomizing 1058 patients to either first-line standard ET followed by *CDK4/6* inhibitor plus ET at progression (strategy A) or first-line *CDK4/6* inhibitor plus ET followed by ET alone at progression (strategy B). The study demonstrated that strategy A was non-inferior to strategy B for PFS2 (defined as time from randomization to progression on second-line therapy or death; median 31.0 vs. 26.7 months; hazard ratio, 1.03; 95% CI, 0.88–1.21), with significantly lower toxicity and healthcare costs in the first-line setting. However, the OS data remain immature. The SONIA trial suggests that delaying *CDK4/6* inhibitor initiation to the second line may be a reasonable strategy for selected patients, particularly in resource-limited settings. In well-resourced settings with unrestricted drug access, upfront combination remains reasonable given the PFS advantage and patient preference for delaying progression, but clinicians should discuss that deferring *CDK4/6* inhibition to the second line does not compromise PFS2 and may reduce cumulative toxicity. For elderly patients or those with low disease burden, an endocrine-first approach may be particularly appropriate. In contrast, in resource-constrained settings where *CDK4/6* inhibitor availability is restricted, the SONIA data provide strong support for initiating endocrine monotherapy and reserving *CDK4/6* inhibitors for progression, thereby maximizing population-level drug impact. For patients with visceral metastases requiring rapid disease control, upfront combination may still be preferred regardless of resource setting, although this subgroup was underrepresented in SONIA [42].

#### 3.2.3. Post-Progression Strategies

After progression on first-line *CDK4/6* inhibitor-based therapy, the optimal sequencing strategy remains an area of active investigation. The phase III post-MONARCH trial (*n* = 368) showed that continuing abemaciclib together with fulvestrant resulted in a longer PFS than fulvestrant alone (median 6.0 vs. 5.3 months; hazard ratio, 0.73; 95% CI, 0.57–0.95; *p* = 0.017) [43]. In contrast, the PACE trial did not demonstrate any advantage to maintaining palbociclib beyond progression (hazard ratio, 1.11; 90% CI, 0.79–1.55) [44]. This discrepancy is likely driven by differences in drug-specific pharmacology, particularly the higher *CDK4* selectivity of abemaciclib and its potentially more favorable pharmacokinetics, which may be especially relevant in drug-resistant settings. Pending direct comparative data, the post-MONARCH trial supported the continuation of abemaciclib after progression, whereas the PACE trial did not support the ongoing use of palbociclib. Additionally, in the MAINTAIN study, switching from palbociclib to ribociclib as the *CDK4/6* inhibitor was linked to improved PFS (5.29 vs. 2.76 months; hazard ratio, 0.57; 95% CI, 0.39–0.95; *p* = 0.006) [43].

Beyond the established *CDK4/6* inhibitors, the mTOR inhibitor everolimus combined with exemestane has demonstrated efficacy in real-world cohorts after *CDK4/6* inhibitor progression [45]. Additionally, dalpiciclib, a next-generation *CDK4/6* inhibitor approved in China, achieved prolonged progression-free survival in combination with fulvestrant in the phase III DAWNA-1 trial [46].

It is important to note that cross-trial comparisons of these post-progression strategies are limited by differences in patient populations, prior therapies, and study designs (Table 1).

**Figure 2:**
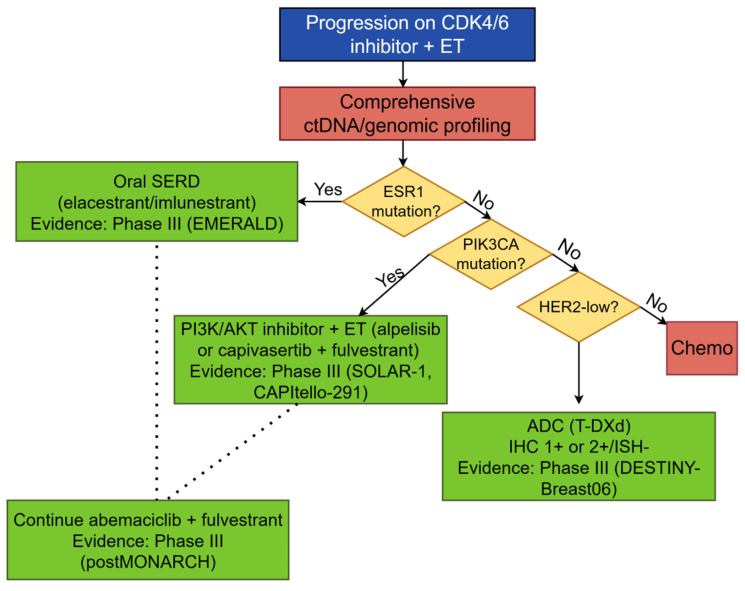
Biomarker-guided treatment algorithm for HR+/HER2− metastatic breast cancer post first-line *CDK4/6* inhibitor + endocrine therapy. Therapy selection stratified by ctDNA *ESR1*, *PIK3CA* and HER2 status. Solid lines = Phase III standard regimens; gray dashed lines = alternative backup strategies. Abbreviations: ADC, antibody–drug conjugate; SERD, selective estrogen receptor degrader.

Systemic treatment strategies after *CDK4/6* inhibitor failure have been comprehensively summarized in a recent systematic review, yet head-to-head prospective randomized trials comparing distinct sequencing regimens are still scarce [47]. For patients with *ESR1* mutations detected via ctDNA or tissue biopsy, oral SERDs (e.g., elacestrant) represent a standard therapeutic option validated by the EMERALD trial [11]. As an alternative strategy, sustained abemaciclib combined with fulvestrant is recommended based on findings from the post-MONARCH trial [43]. For *PIK3CA*-altered tumors, alpelisib plus fulvestrant is the preferred regimen [41]. If no targetable genomic aberrations are detected, switching from palbociclib to ribociclib yields prolonged PFS as demonstrated in the MAINTAIN trial [48]; antibody–drug conjugates including trastuzumab deruxtecan and datopotamab deruxtecan also deliver clinical benefits regardless of mutation profiles. For patients with rapid symptomatic disease progression, chemotherapy (capecitabine or eribulin) remains the standard choice (Table 1). Figure 2 illustrates a biomarker-driven treatment algorithm for patients progressing on first-line *CDK4/6* inhibitor plus endocrine therapy, which integrates ctDNA molecular profiling and evidence-based sequential regimens. This framework stratifies subsequent therapies by actionable genomic changes (*ESR1* mutations, *PIK3CA* alterations, HER2-low status) and labels each intervention with corresponding evidence tiers (Phase III RCT, Phase II, or investigational).

It is important to emphasize that this treatment algorithm represents a proposed clinical framework based on currently available evidence and should be applied within the context of multidisciplinary tumor board discussion, patient preferences, and clinical trial availability. The sequencing of therapies after *CDK4/6* inhibitor progression remains an active area of investigation, and treatment decisions should be individualized based on the biomarker profile, prior treatment history, toxicity considerations, and the pace of disease progression. For patients without actionable mutations, chemotherapy (e.g., capecitabine, eribulin) remains the standard of care, while enrollment in clinical trials evaluating novel strategies (including *CDK4*-selective inhibitors, PROTAC-based degraders, and autophagy inhibitors) should be encouraged whenever feasible.

### 3.3. Pharmacological Distinctions Among Approved CDK4/6 Inhibitors

Although palbociclib, ribociclib, and abemaciclib share the same primary target, they exhibit important pharmacological differences that may contribute to their distinct clinical profiles. In enzymatic assays, abemaciclib is the most potent *CDK4/6* inhibitor, with IC50 values of 2 nM for *CDK4*/cyclin D1 and 9.9 nM for *CDK6*/cyclin D3, yielding a *CDK4*:*CDK6* selectivity ratio of approximately 1:5 (or 14-fold greater potency for *CDK4* over *CDK6*) [49]. Palbociclib displays comparable potency against *CDK4* (IC50 9–11 nM) and *CDK6* (IC50 15 nM), with a *CDK4*:*CDK6* ratio of approximately 1:1.5 [9]. Ribociclib shows intermediate selectivity, with IC50 values of 10 nM for *CDK4* and 39 nM for *CDK6* (ratio 1:4) [9].

These biochemical differences have important clinical implications. The higher *CDK4* selectivity of abemaciclib may explain its lower incidence of grade 3–4 neutropenia (~22%) compared with palbociclib (~66%) and ribociclib (~60%), as *CDK6* plays a critical role in hematopoietic stem cell differentiation [50]. Conversely, abemaciclib’s additional inhibition of *CDK9* (IC50 ~ 57 nM) and other kinases may contribute to its distinct toxicity profile, particularly the higher incidence of diarrhea (81% vs. 26–35% for palbociclib and ribociclib) and gastrointestinal adverse events [51]. Unlike palbociclib and ribociclib, which require intermittent dosing (21 days on/7 days off) to allow neutrophil recovery, abemaciclib can be administered continuously due to its more favorable hematologic safety profile [52].

Pharmacokinetic parameters also differ among the three agents. Palbociclib has a mean half-life of 29 h and is primarily metabolized by CYP3A4 and SULT2A1; food intake modestly increases its absorption [53]. Ribociclib has a similar half-life of 32 h and requires dose reduction when co-administered with strong CYP3A4 inhibitors [53]. Abemaciclib has a shorter half-life of 18.3 h, necessitating twice-daily administration, and is also hepatically metabolized by CYP3A4 [53]. Abemaciclib is the only agent with demonstrated blood–brain barrier penetration, with brain concentrations approximately 96-fold higher than the *CDK4* IC50, leading to interest in its potential activity against CNS metastases [54]. These pharmacological distinctions support the concept that *CDK4/6* inhibitors should not be regarded as interchangeable agents with a uniform class effect, but rather as distinct therapeutic entities with agent-specific efficacy, toxicity, and resistance profiles (Table 2).

Common toxicities include neutropenia (palbociclib/ribociclib, ~60% grade 3–4; abemaciclib, ~25%), fatigue, and diarrhea, with the latter occurring more frequently with abemaciclib. Ribociclib necessitates routine monitoring of liver function due to grades 3–4 transaminase elevation in approximately 10–15% of patients [29,44]. Dose adjustments are frequently required; however, proactive toxicity management allows the therapy to be maintained. Abemaciclib was associated with increased risks of venous thromboembolism (5.0% vs. 2.7% with placebo) and interstitial lung disease (2.8% vs. 0.5%) [39]. The health-related quality-of-life data indicated overall tolerability, although diarrhea and fatigue were more pronounced than in those treated with ET alone [30]. A systematic review reported that the addition of *CDK4/6* inhibitors to radiotherapy results in only modest additional toxicity [55]. Economic constraints restrict worldwide availability; cost-effectiveness evaluations in the US demonstrate incremental cost-effectiveness ratios that surpass commonly accepted thresholds [56]. Management strategies for toxicities in special groups (e.g., older adults, Asian patients, and those with organ dysfunction) are outlined in Table 3.

Beyond the aforementioned special populations, premenopausal patients and men with HR+/HER2− breast cancer represent another distinct clinical subgroup requiring tailored treatment considerations. In premenopausal patients, ovarian function suppression combined with a *CDK4/6* inhibitor is an effective strategy, as shown in the MONALEESA-7 trial, which reported improved PFS and OS with ribociclib plus tamoxifen or an aromatase inhibitor, both given with ovarian function suppression [57]. In male breast cancer, evidence is sparse; current guidance largely relies on data from studies in women, and identical dosing and monitoring are generally recommended. Because pregnancy is contraindicated during *CDK4/6* inhibitor treatment, options for fertility preservation should be addressed before therapy begins [28].

## 4. Mechanisms of Response and Resistance

The pharmacological distinctions detailed above—including differential *CDK4/6* selectivity, unique toxicity spectra and variable blood–brain barrier permeability—explain variable resistance patterns across the three approved *CDK4/6* agents. To move beyond simple mechanistic cataloging toward clinically actionable risk stratification, we introduce a unified resistance taxonomy to organize all subsequent molecular and biomarker analysis.

At the outset of this section, we define our resistance taxonomy and its biomarker boundaries, as this framework underpins all further discussion. Target-driven resistance refers to alterations that directly compromise *CDK4/6* inhibitor binding or downstream pathway dependence, including *ESR1* mutations (detectable by ctDNA), *PIK3CA*/AKT/*PTEN* alterations, *CDK6* amplification, and CCND1 overexpression. These mechanisms retain reliance on the cyclin D–*CDK4/6*–RB axis yet evade pharmacological blockade. Bypass-driven resistance, by contrast, involves complete loss of pathway dependence, most notably via *RB1* loss, *CCNE1* amplification, or FGFR/MEK pathway activation, enabling tumor proliferation independent of *CDK4/6* activity. A critical clinical distinction is that target-driven alterations correspond to actionable therapeutic options (e.g., *ESR1* mutations → fulvestrant/oral SERDs; *PIK3CA* mutations → alpelisib), while bypass-driven resistance currently lacks approved targeted therapies and generally necessitates chemotherapy or clinical trial enrollment. Table 4 summarizes all resistance mechanisms, paired with their associated biomarkers and clinical implementability status.

**Figure 3:**
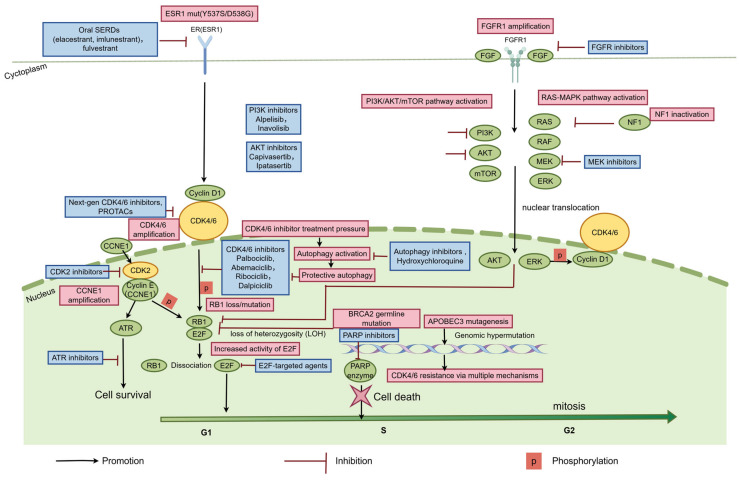
Mechanisms of *CDK4/6* Inhibitor Resistance in HR+/HER2− Breast Cancer and Targeted Therapeutic Strategies.

We then proceed to dissect predictive biomarkers, followed by separate analysis of primary and acquired resistance phenotypes (Figure 3).

### 4.1. Biomarkers

Distinguishing prognostic from predictive biomarkers is critical for interpreting *CDK4/6* inhibitor clinical data. Prognostic markers reflect intrinsic tumor outcomes regardless of therapy, while predictive biomarkers stratify patients by treatment responsiveness. To date, most available biomarkers carry only prognostic utility, with few robust predictive signatures validated for *CDK4/6* inhibition.

#### 4.1.1. Prognostic Biomarkers (Tissue-Based)

The loss of *RB1* is the most powerful driver of primary resistance. In patient-derived xenograft (PDX) models, *RB1*-mutant tumors showed no response (0%) compared to a 50% response rate in *RB1* wild-type tumors (*p* < 0.001) [10]. Ki-67 suppression serves as a dynamic pharmacodynamic marker. Serum thymidine kinase 1 activity closely mirrors Ki-67 suppression (κ = 0.76) and predicts CCCA [65]. Low progesterone receptor expression (<20%) is an independent predictor of shorter PFS (hazard ratio, 2.84; 95% CI, 1.08–7.45; *p* = 0.043) [66].

#### 4.1.2. Predictive Biomarkers (Liquid Biopsy)

Changes in ctDNA levels are prognostic factors. In the PALOMA-3 trial, early decreases in *PIK3CA* ctDNA were strongly associated with later PFS outcomes (hazard ratio, 3.94; 95% CI, 1.72–9.01; *p* = 0.0013) [65]. Therefore, *ESR1* mutations are clinically feasible. In the PADA-1 trial, among patients who developed rising blood *ESR1* mutations while on aromatase inhibitor plus palbociclib, an early switch to fulvestrant plus palbociclib extended PFS from 5.7 to 11.9 months (stratified hazard ratio, 0.61; 95% CI, 0.43–0.86; *p* = 0.004) [12]. A systematic review further showed that ctDNA positivity during adjuvant therapy was associated with inferior iDFS (hazard ratio, 2.5; 95% CI, 1.9–3.3) [67].

The biomarkers discussed above serve not only as prognostic or predictive tools but also as indicators of specific resistance phenotypes. In the following sections, we apply the target-driven vs. bypass-driven resistance taxonomy to dissect primary and acquired resistance mechanisms, linking each molecular alteration to its clinical actionability (Table 4).

### 4.2. Primary Resistance

Primary resistance is largely mediated by the *RB1*–E2F axis. Loss-of-function mutations in *RB1* render cells unresponsive. Amplification of *CCNE1* (cyclin E1) enables cells to circumvent the G1/S checkpoint via *CDK2*. In PDX models, tumors with *CCNE1* amplification exhibited only a modest response to palbociclib (10% tumor growth inhibition vs. 70% in non-amplified tumors) [10].

The activation of the PI3K/AKT/mTOR signaling cascade is a key mechanism. *PIK3CA* mutations (present in ~40% of HR+/HER2− breast cancers) decrease drug sensitivity [41]. The presence of *PIK3CA* mutations in ctDNA has been associated with reduced PFS (7.44 vs. 12.9 months, *p* = 0.01) [58]. Beyond *PIK3CA* mutations, other alterations in the PI3K/AKT/mTOR pathway contribute to bypass-driven resistance. *PTEN* loss (occurring in approximately 10–15% of HR+/HER2− breast cancers) relieves negative regulation of PI3K signaling, leading to constitutive AKT activation that promotes cell cycle progression independent of *CDK4/6* activity. Similarly, activating *AKT1* mutations (E17K, present in 3–5% of cases) directly phosphorylate downstream targets, including p21CIP1 and p27KIP1, thereby reducing their inhibitory effects on cyclin E–*CDK2* complexes and enabling G1/S transition despite *CDK4/6* inhibition. These alterations represent canonical bypass-driven resistance mechanisms because they operate downstream of or parallel to the *CDK4/6*–RB axis, rendering *CDK4/6* inhibition insufficient for cell cycle arrest. Preclinical studies have demonstrated that *PTEN*-deficient or *AKT1*-mutant breast cancer cell lines exhibit reduced sensitivity to palbociclib, and combination strategies targeting PI3K/AKT (such as capivasertib) have shown promise in overcoming this resistance phenotype. Inactivating *NF1* mutations (~8.1% of advanced breast cancers) promote resistance through hyperactivation of the RAS–MAPK pathway [59]. In addition, *FGFR1* amplification (10–15%) is associated with shorter PFS in the MONALEESA-2 trial (hazard ratio, 1.5; 95% CI, 1.0–2.3; *p* = 0.03) [60].

### 4.3. Acquired Resistance

*ESR1* mutations affecting codons 380 and 536–538 arise in 25–40% of patients following aromatase inhibitor treatment and represent the most common mechanism of acquired resistance. Despite this, *CDK4/6* inhibitors remain effective in *ESR1*-mutant disease, as demonstrated in the PADA-1 trial [12] (Table 4).

In contrast, *CDK6* amplification constitutes an on-target resistance mechanism; in PDX models, *CDK6*-amplified tumors showed a two-fold increase in the half-maximal inhibitory concentration (IC_50_) for palbociclib [10]. *APOBEC3*-driven mutagenesis further accelerates resistance, with *APOBEC3*-dominant mutational signatures independently correlating with shorter PFS (hazard ratio, 1.5; 95% CI, 1.2–1.8; *p* < 0.001) [62]. In addition, germline *BRCA2* mutations frequently co-occur with *RB1* loss of heterozygosity linked to 13q and are associated with shorter PFS (median 9.0 vs. 13.7 months; multivariate hazard ratio, 2.28; 95% CI, 1.52–3.39; *p* = 4.9 × 10^−5^) [63] (Table 4).

## 5. Novel Strategies and Future Directions

The resistance mechanisms catalogued in Section 4 are not merely academic categories; they point toward specific therapeutic vulnerabilities. The practical question is which of these opportunities are sufficiently mature to change clinical practice today, and which remain experimental hypotheses awaiting prospective validation. Building on the target-driven/bypass-driven resistance framework established in Chapter 4, we further stratify each resistance subtype based on clinical implementability rather than molecular features alone. As argued in our third proposition, the most impactful near-term advances will stem from optimized combinatorial regimens rather than novel single agents. Here, we review several promising research directions, including next-generation degraders, oral SERDs, and ADCs, alongside strategies targeting adaptive resistance pathways.

### 5.1. Next-Generation CDK4/6-Targeted Agents

PROteolysis-Targeting Chimeras (PROTACs) represent an emerging approach that drives the degradation of *CDK4/6*, thereby abolishing both their enzymatic activity and scaffolding functions—a mechanism distinct from traditional enzymatic inhibition. The biological rationale for PROTAC development in this setting is compelling: by completely removing the target protein rather than merely inhibiting its kinase activity, PROTACs may overcome resistance mediated by *CDK6* amplification or activating mutations that reduce drug binding affinity. Pal-pom and rib-pom (palbociclib and ribociclib conjugated to pomalidomide) have demonstrated DC_50_ values of 12.9–97 nM in preclinical models [14]. *CDK6*-selective PROTACs, such as YX-2-107, achieve DC_50_ values of approximately 4 nM while sparing normal hematopoietic progenitor cells [15]. Although promising, all PROTAC-based approaches remain in preclinical or very early-phase development, with unresolved challenges in oral bioavailability, tissue penetration, off-target effects, and long-term safety. Various PROTAC constructs recruiting cereblon, VHL or IAP E3 ligases have displayed anti-tumor activity and remain in early-stage clinical testing for breast cancer [14,15].

In parallel, *CDK4*-selective inhibitors seek to enhance the therapeutic index by avoiding *CDK6* inhibition, thereby limiting adverse hematologic effects. TQB3616 is a new *CDK4*-selective inhibitor that has advanced to phase III clinical trials in HR+/HER2− breast cancer [16].

### 5.2. Novel Endocrine Therapies (Oral SERDs)

Oral SERDs have several advantages over intramuscular fulvestrants, including improved bioavailability and greater convenience [68]. Elacestrant, the first oral SERD approved by the FDA, demonstrated superior PFS compared with standard ET in the EMERALD trial (overall hazard ratio, 0.70; 95% CI, 0.55–0.88; *p* = 0.002; *ESR1*-mutant subgroup hazard ratio, 0.55; 95% CI, 0.39–0.77; *p* = 0.0005) [11]. In the EMBER-3 trial, the brain-penetrant oral SERD imlunestrant achieved a median PFS of 5.5 months vs. 3.8 months in patients with *ESR1*-mutant tumors (restricted mean survival time difference 2.6 months, *p* < 0.001). The addition of abemaciclib further extended PFS to 9.4 months compared with 5.5 months (hazard ratio, 0.57; 95% CI, 0.44–0.73; *p* < 0.001) [13].

### 5.3. Antibody–Drug Conjugates (ADCs)

ADCs represent an emerging therapeutic option for selected patients with HR+/HER2− breast cancer after progression on *CDK4/6* inhibitors, although their use is contingent upon specific eligibility criteria. In the DESTINY-Breast06 trial, trastuzumab deruxtecan (T-DXd) improved PFS compared with standard chemotherapy (median 13.2 vs. 8.1 months; hazard ratio, 0.62; 95% CI, 0.52–0.75; *p* < 0.001) in patients with HER2-low (IHC 1+ or 2+/ISH−) metastatic breast cancer, many of whom had prior *CDK4/6* inhibitor exposure [69]. It is important to note that T-DXd is approved specifically for HER2-low disease, and its efficacy in HER2-zero (IHC 0) populations remains investigational. Additionally, the risk of interstitial lung disease/pneumonitis (occurring in approximately 12% of patients) requires careful patient selection and monitoring. In the TROPION-Breast01 trial, datopotamab deruxtecan (Dato-DXd) extended PFS to 6.9 months compared with 4.9 months (hazard ratio, 0.63; 95% CI, 0.52–0.76; *p* < 0.0001) [70]. Ongoing studies, such as the TROPION-Breast05 trial, are investigating combining ADCs with immunotherapy [71]. In addition, exploratory analyses from the TROPiCS-02 study indicated that patients with DNA damage repair defects may derive greater benefit from sacituzumab govitecan [72].

### 5.4. Targeting the Tumor Microenvironment and Adaptive Resistance Pathways

*CDK4/6* inhibitors alter the tumor immune microenvironment and increase programed death-ligand 1 expression [73]. However, combination strategies involving immunotherapy are limited by toxicity. The CheckMate 7A8 trial (neoadjuvant nivolumab + palbociclib + anastrozole) was halted prematurely because 44.4% of patients developed grade 3–4 hepatotoxicity [74]. In the NEWFLAME study (nivolumab + abemaciclib + ET), clinical activity with an acceptable safety profile was observed, although liver toxicity remained a notable issue [75]. It remains unclear whether these discrepancies reflect intrinsic differences between the drugs (palbociclib in CheckMate 7A8 vs. abemaciclib in NEWFLAME) or are driven by the trial design and context (neoadjuvant vs. metastatic setting and patient population). Pending head-to-head trials, we advise against routine immunotherapy with *CDK4/6* inhibitor combinations outside clinical trials. The hepatotoxicity signal is real and its benefits remain unproven (Figure 3).

In addition to immune modulation, adaptive resistance through autophagy and activation of the MAPK pathway provides further therapeutic opportunities. Blocking autophagy can resensitize tumors to *CDK4/6* inhibition. A phase I/II study of hydroxychloroquine plus high-dose palbociclib in heavily pretreated *CDK4/6* inhibitor-resistant MBC reported an objective response rate of 41.4% and 6-month clinical benefit rate of 90%, as highlighted in a recent review [64]. Concurrent MEK and *CDK4/6* inhibition is also being tested, particularly in cancers with MAPK pathway activation; preclinical work in *KRAS*-mutant models supports this strategy [76]. These therapeutic approaches are currently being explored in clinical trials (Figure 3).

### 5.5. Central Nervous System (CNS) Metastases

Abemaciclib demonstrates blood–brain barrier penetration, which has generated interest in its potential activity against CNS metastases. In a small phase II trial of 58 patients with HR+/HER2− brain metastases, the intracranial clinical benefit rate was 24% (95% CI, 13.1–35.2), and median OS was 12.5 months; abemaciclib concentrations in brain metastases were 96-fold higher than the *CDK4* IC_50_ and 19-fold higher than the *CDK6* IC_50_ [54]. However, evidence supporting *CDK4/6* inhibitor activity in CNS disease remains limited to small, single-arm studies, and their role in the prevention or treatment of brain metastases is currently investigational. Large prospective trials specifically designed to evaluate CNS efficacy are needed to define the clinical utility of *CDK4/6* inhibitors in this setting. In the EMBER-3 study, the 12-month cumulative incidence of CNS progression was 1.5% in the imlunestrant arm vs. 6.7% with standard therapy (cause-specific hazard ratio, 0.18; 95% CI, 0.04–0.90) [13], supporting a potential CNS-protective effect.

### 5.6. Future Directions

Several emerging technologies are under investigation but remain at early stages of development. Machine learning-based adaptive treatments, radiomics for predicting responses, and integrated multi-omics approaches have shown preliminary promise but require validation in prospective clinical trials. One proof-of-concept machine learning model predicted 12-month PFS with an area under the receiver operating curve of 0.78 in a retrospective cohort of patients receiving *CDK4/6* inhibitors [17]. Radiomic signatures derived from computed tomography have reported 82% accuracy for predicting early treatment responses in small exploratory studies [18]. It is important to emphasize that these approaches are currently investigational and have not yet been integrated into routine clinical practice or guidelines. The PADA-1 strategy of ctDNA-guided treatment switching illustrates real-time therapeutic optimization [12]. Current trials are investigating ctDNA-based strategies for treatment discontinuation (for example, the SERENA-6 trial, which assesses early switching to camizestrant upon ctDNA detection of emerging *ESR1* mutations in the absence of radiological progression [77]). Simultaneously, combinations of *CDK4*-selective inhibitors with oral SERDs are being tested in phase III studies to enhance their efficacy while limiting toxicity. Targeting *APOBEC3*-driven mutagenesis [62] and cancer stem cell-related pathways [78] remains a key focus in preclinical research. Bioinformatics pipelines and multi-omics platforms (e.g., MOFA and mixOmics) support individualized treatment strategies [79]. Next-generation agents (such as PROTACs and highly selective inhibitors) and rational therapeutic combinations (including ADCs, SERDs, and immunotherapy) are expected to further refine and personalize *CDK4/6*-directed therapy [80].

## 6. Conclusions

While machine learning, radiomics and multi-omic analytical tools outlined in Section 5.6 remain largely investigational, this review constructs a unified clinical framework centered on three translational challenges: divergent efficacy between adjuvant and metastatic settings, SONIA trial-informed therapeutic sequencing, and biomarker-guided resistance stratification. Despite the paradigm shift brought by *CDK4/6* inhibitors in HR+/HER2− breast cancer, critical gaps remain when converting clinical trial results into standardized real-world treatment sequencing.

In the adjuvant setting, abemaciclib and ribociclib lower recurrence risk for high-risk patients, while palbociclib yields no such benefit, consistent with our first core proposition. Interim analyses show iDFS gains have not yet translated to consistent overall survival (OS) improvements, implying sustained follow-up (≥3–4 years) may be necessary to detect meaningful OS separation. Clinicians should inform patients that adjuvant *CDK4/6* inhibition mitigates recurrence, though long-term OS benefits remain unconfirmed to date. In the metastatic setting, all three agents prolonged PFS in the first line, and ribociclib and potentially abemaciclib extended OS. However, the SONIA trial has fundamentally challenged the assumption that upfront *CDK4/6* inhibition followed by endocrine therapy alone at progression is superior to an endocrine-first strategy, with *CDK4/6* inhibition reserved for the second line. Strategy A (first-line ET, *CDK4/6* inhibitor at progression) was non-inferior to strategy B (upfront *CDK4/6* inhibitor) for PFS2, with significantly lower toxicity and healthcare costs—findings that support considering endocrine-monotherapy sequencing in selected patients, particularly in resource-limited settings where drug accessibility is a major barrier.

Looking ahead, the most impactful near-term advance will arise not from novel inhibitors but from rational sequencing informed by the SONIA paradigm. In well-resourced settings with broad access to *CDK4/6* inhibitors, upfront combination remains reasonable given the PFS advantage and patient preference for delaying progression; however, clinicians should discuss with patients that deferring *CDK4/6* inhibition to the second line does not compromise PFS2 and may reduce cumulative toxicity. In resource-limited settings where *CDK4/6* inhibitor availability is restricted or cost-prohibitive, the SONIA data provide strong support for initiating endocrine monotherapy and reserving *CDK4/6* inhibitors for progression, thereby maximizing the population-level impact of these drugs. This distinction—between optimal sequencing in ideal conditions and pragmatic sequencing where access is constrained—should be central to future guidelines. For patients with acquired resistance, treatment selection should be guided by the biomarker-directed framework outlined in this review, integrating molecular profiling to identify actionable alterations while prioritizing clinical trial enrollment for mechanisms lacking established therapies. Future research should focus on prospective validation of biomarker-driven algorithms and the development of accessible diagnostic platforms to enable global implementation of precision sequencing strategies.

## Figures and Tables

**Table 1:** Key Clinical Trials of *CDK4/6* Inhibitors in HR+/HER2− Metastatic Breast Cancer.

Trial	Phase	Line	Regimen	Sample Size	Median PFS (months)	HR (95% CI)	OS Benefit and Additional Findings
PALOMA-2 [3]	III	First-line	Palbociclib + Letrozole vs. Placebo + Letrozole	666 (444 vs. 222)	24.8 vs. 14.5	0.58 (0.46–0.72)	OS immature; ORR 42.1% vs. 34.7% (*p* = 0.06); CBR 84.9% vs. 70.3% (*p* < 0.001); median DoR 22.5 vs. 16.8 mo
MONALEESA-2 [4]	III	First-line	Ribociclib + Letrozole vs. Placebo + Letrozole	668 (334 vs. 334)	25.3 vs. 16.0	0.56 (0.43–0.72)	Median OS 63.9 vs. 51.4 mo (HR = 0.76, *p* = 0.008); time to first chemo 50.6 vs. 38.9 mo (HR = 0.74)
MONARCH 3 [5]	III	First-line	Abemaciclib + NSAI vs. Placebo + NSAI	493 (328 vs. 165)	28.2 vs. 14.8	0.54 (0.41–0.72)	Median OS 66.8 vs. 53.7 mo (Δ13.1 mo); HR = 0.804 (*p* = 0.066); chemo-free survival 46.7 vs. 30.6 mo (HR = 0.693, *p* = 0.001)
postMONARCH [43]	III	Post-*CDK4/6*i	Abemaciclib + Fulvestrant vs. Placebo + Fulvestrant	368 (182 vs. 186)	6.0 (5.6–8.6) vs. 5.3 (3.7–5.6)	0.73 (0.57–0.95)	OS immature; 6-mo PFS 50% vs. 37%; ORR 17% vs. 7% (*p* = 0.015); benefit across *ESR1*/*PIK3CA* subgroups
PACE [44]	II	Post-*CDK4/6*i + AI	Fulvestrant vs. Fulvestrant + Palbociclib vs. Fulvestrant + Palbociclib + Avelumab	220 (55/111/54)	4.8/4.6/8.1	Palbo + fulv vs. fulv: 1.11 (0.79–1.55); +avelumab vs. fulv: 0.75 (0.50–1.12)	Median OS 27.5, 24.6, 42.5 mo (HR for triplet vs. fulv 0.68 [90% CI 0.40–1.15]); ORR 7.3%, 9.0%, 13.0%; immune AEs in 52.8% of triplet arm

Abbreviations: AI, aromatase inhibitor; CBR, clinical benefit rate; DoR, duration of response; NSAI, nonsteroidal aromatase inhibitor; ORR, objective response rate; OS, overall survival; PFS, progression-free survival.

**Table 2:** Comparative Pharmacology of Approved *CDK4/6* Inhibitors.

Parameter	Palbociclib	Ribociclib	Abemaciclib
*CDK4* IC_50_ (nM)	9–11	10	2
*CDK6* IC_50_ (nM)	15	39	9.9
*CDK9* IC_50_ (nM)	NR	NR	57
*CDK4*:*CDK6* selectivity ratio	1:1.5	1:4	1:5 (14-fold for *CDK4*)
Half-life (hours)	29	32	18.3
Dosing schedule	125 mg daily, 21/28 days	600 mg daily, 21/28 days	150 mg twice daily, continuous
Route of metabolism	Hepatic (CYP3A4, SULT2A1)	Hepatic (CYP3A4)	Hepatic (CYP3A4)
Blood–brain barrier penetration	Limited	Limited	Demonstrated
Grade 3–4 neutropenia	~66%	~60%	~22%
Grade 3–4 diarrhea	~1%	~1%	~9%

Abbreviations: NR, not reported; IC50, half-maximal inhibitory concentration. Data compiled from [9,49,50,51,53].

**Table 3:** Clinical Considerations in Special Populations for *CDK4/6* Inhibitor Therapy.

Population	Key Clinical Considerations	Supporting Evidence	References
Elderly (≥70 years)	Higher grade 3–4 toxicities (45% vs. 30%); need individualized dosing; comparable efficacy with monitoring	Pooled analysis: median PFS 18.6 vs. 10.9 mo (HR = 0.56, *p* < 0.001). Real-world (*n* = 206): median PFS 18.4 mo	[28,34]
Asian populations	Efficacy similar to Western; consistent PFS benefit	Korean (*n* = 1115): HR = 0.64 (0.55–0.75), *p* < 0.001. Japanese (*n* = 104): median PFS 19.3 mo	[35,36]
Hepatic impairment	Ribociclib: monitor LFTs (10–15% grade 3–4 hepatotoxicity). Abemaciclib: lower hepatotoxicity, preferred if baseline dysfunction	Grade 3–4 LFT elevations: ribociclib 10–15%, abemaciclib <5%. Dose adjustment per severity	[28,29]
Renal impairment	No dose adjustment for mild-to-moderate impairment; limited data for severe	Pharmacokinetics: no meaningful exposure changes	[28]
CNS metastases	Abemaciclib penetrates BBB; offers treatment option	Phase II (*n* = 58): iCBR 24% (95% CI 13.1–35.2); median OS 12.5 mo; brain concentrations 96× *CDK4* IC_50_, 19× *CDK6* IC_50_	[54]
Drug accessibility	Cost barriers limit global access; ICER exceeds willingness-to-pay	US cost-effectiveness: ICER $457,578/QALY. Global surveys: 30% in low-income countries cannot access	[28]
Adherence & discontinuation	Discontinuation rates 31–42%; adherence critical; proactive toxicity management	Discontinuation: PALLAS 42%, monarchE 31%, NATALEE 36%. NATALEE HRQoL stable; compliance 93–97%	[7,28,35]

Abbreviations: BBB, blood–brain barrier; CNS, central nervous system; HRQoL, health-related quality of life; iCBR, intracranial clinical benefit rate; ICER, incremental cost-effectiveness ratio; IC_50_, half-maximal inhibitory concentration; LFT, liver function test; QALY, quality-adjusted life year.

**Table 4:** Mechanisms of Resistance to *CDK4/6* Inhibitors: Biomarkers and Clinical Actionability.

Resistance Category	Key Molecular Alterations/Mechanisms	Core Evidence	Representative Clinical/Translational Findings	Potential Intervention Strategies
Primary Resistance	*RB1* loss-of-function mutation/deletion; *CCNE1* amplification	*RB1* is essential for *CDK4/6* inhibitor response; *RB1*-null tumors are fully resistant. *CCNE1* amplification bypasses G1/S checkpoint via *CDK2* [10]	0% response to palbociclib in *RB1*-mutant PDX models; *CCNE1* amplification linked to minimal tumor growth inhibition [10]	*CDK2* inhibitors; E2F-targeted agents; ATR inhibitors
	*PIK3CA*/*AKT*/*PTEN* pathway activation	PI3K pathway activation drives intrinsic resistance; ctDNA *PIK3CA* mutations correlate with shorter PFS [58]	*PIK3CA*-mutant patients have shorter PFS (7.44 vs. 12.9 months) on *CDK4/6* inhibitors [58]	PI3K/AKT inhibitors + ET [47]
	*NF1* inactivation	*NF1* loss hyperactivates RAS-MAPK and causes endocrine resistance; *CDK4/6* inhibitors may overcome this [59]	*NF1* mutations enriched in ~8.1% of advanced breast cancer [59]	MEK inhibitors; SOS1 inhibitors
	*FGFR1* amplification	FGFR signaling mediates resistance via bypass pathway [60]	*FGFR1* amplification linked to shorter PFS in MONALEESA-2 (HR = 1.5, *p* = 0.03) [60]	FGFR inhibitors; pan-RTK inhibitors
Acquired Resistance	*ESR1* mutations (Y537S, D538G)	Most common acquired mechanism; 25–40% after AI exposure; detectable by ctDNA [12,61]	Early ctDNA *ESR1* mutation predicts progression; switching to fulvestrant improves PFS [12]	Oral SERDs (elacestrant, imlunestrant) [47]; fulvestrant +*CDK4/6* inhibitors
	*CDK6* amplification	On-target resistance via increased *CDK6* expression; reduces drug binding [10]	Two-fold higher IC_50_ for palbociclib in *CDK6*-amplified PDXs [10]	Next-gen *CDK4/6* inhibitors; PROTACs
	*APOBEC3* mutagenesis	*APOBEC3* mutational signature accelerates resistance acquisition [62]	*APOBEC3*-high tumors show shorter PFS (HR = 1.5, *p* < 0.001) [62]	*APOBEC3* inhibitors; DDR modulators
	*BRCA2* germline mutation	*BRCA2* mutations frequently co-occur with *RB1* LOH (13q linkage) [63]	Germline *BRCA2* associated with shorter PFS (median 9.0 vs. 13.7 months) [63]	PARP inhibitors; platinum-based chemo
Adaptive/Pathway Bypass	Autophagy activation	*CDK4/6* inhibitors induce cytoprotective autophagy via *PTEN*-PI3K-AKT signaling [64]	High-dose *CDK4/6* inhibitors + hydroxychloroquine restores sensitivity in resistant patients [64]	Hydroxychloroquine; autophagy inhibitors
	RAS-MAPK hyperactivation	Alternative pathway bypasses cell cycle control [59]	Confirmed in preclinical models and patient samples [59]	MEK + *CDK4/6* inhibitor combination

## Data Availability

No new data were created or analyzed in this study. Data sharing is not applicable to this article.
